# Low oxytocin levels are broadly associated with more pronounced psychopathology in anorexia nervosa with primarily restricting but not binge/purge eating behavior

**DOI:** 10.3389/fendo.2022.1049541

**Published:** 2023-01-31

**Authors:** Franziska Plessow, Francesca Galbiati, Kamryn T. Eddy, Madhusmita Misra, Karen K. Miller, Anne Klibanski, Anna Aulinas, Elizabeth A. Lawson

**Affiliations:** ^1^ Neuroendocrine Unit, Department of Medicine, Massachusetts General Hospital and Harvard Medical School, Boston, MA, United States; ^2^ Eating Disorders Clinical and Research Program, Department of Psychiatry, Massachusetts General Hospital and Harvard Medical School, Boston, MA, United States; ^3^ Division of Pediatric Endocrinology, Massachusetts General Hospital for Children and Harvard Medical School, Boston, MA, United States

**Keywords:** anorexia nervosa, binge/purge behavior, dietary restriction, oxytocin, psychopathology

## Abstract

**Objective:**

Anorexia nervosa (AN) is commonly associated with depression, anxiety, and deficits in socioemotional functioning. Basal levels of oxytocin, a neurohormone with antidepressant, anxiolytic, and prosocial properties, are low in women with AN. However, the relationship between oxytocin and psychopathology of AN/atypical AN has not been examined in individuals with primarily food restriction (AN/AtypAN-R) or those with restriction plus binge/purge behaviors (AN/AtypAN-BP) alone, which is important to further elucidate the neurobiology of different AN presentations. We investigated whether oxytocin levels are related to eating, affective, and socioemotional psychopathology in women with AN/AtypAN-R and separately AN/AtypAN-BP.

**Methods:**

In a cross-sectional study of 53 women with low-weight AN or atypical AN based on DSM-5 (AN/AtypAN-R: n=21, AN/AtypAN-BP: n=32), we obtained fasting serum oxytocin levels and self-report measures of psychopathology, including the Eating Disorder Examination–Questionnaire (EDE-Q), Beck Depression Inventory-IA (BDI), State-Trait Anxiety Inventory (STAI), and Toronto Alexithymia Scale (TAS-20).

**Results:**

In individuals with AN/AtypAN-R, oxytocin levels were negatively associated with eating psychopathology (EDE-Q Global Score: r=-0.49, p=0.024), depressive and anxiety symptoms (BDI Total Score: r=-0.55, p=0.009; STAI Trait Score: r=-0.63, p=0.002), and socioemotional symptoms (TAS-20 Difficulty Identifying Feelings Score: r=-0.49, p=0.023). In contrast, in those with AN/AtypAN-BP oxytocin levels were negatively associated with depressive symptoms only (BDI Total Score: r=-0.52, p=0.049).

**Conclusions:**

These findings support the notion that AN/AtypAN-R and AN/AtypAN-BP might have divergent underlying neurobiology. Understanding these differences is crucial to develop targeted treatments for a population with high levels of chronicity, for which no specific pharmacological treatments are currently available.

**Clinical trial registration:**

https://clinicaltrials.gov, identifier: NCT01121211.

## 1 Introduction

Anorexia nervosa (AN), a psychiatric disorder with a prime onset period in adolescence and early adulthood, manifests with different clinical presentations, calling for treatments based on carefully developed pathophysiological models. Its core clinical features include a distorted body image, intense fear of gaining weight, and food restriction despite a low body mass index (BMI) (1). It is also characterized by the common occurrence of comorbid symptoms, including depressive and anxiety symptoms and deficits in socioemotional functioning (2–4). AN has two major clinical presentations; (i) predominantly food restriction, or (ii) food restriction combined with cycles of binge eating and/or purging behaviors. AN is often treatment-refractory, and novel treatments are needed to improve outcomes. Investigating the neurobiological mechanisms underlying restricting and binge/purge presentations could inform urgently needed individualized treatment strategies.

The neurohormone oxytocin affects food intake as well as cognitive, emotional, and social functioning and might play a role in the pathophysiology of AN spanning ED pathology and associated depressive and anxiety symptoms and impairments in socioemotional functioning (5). Prior studies of females with AN demonstrate oxytocin deficiency in the setting of chronic starvation (6–9). These findings are complemented by studies showing that weight-restored individuals with a history of AN have lower basal oxytocin levels than healthy controls, suggesting chronic alteration of oxytocin signaling (2, 10). Furthermore, our group has previously shown that in women with restored weight but persistent symptoms of disordered eating, fasting oxytocin levels were associated with greater ED pathology and more pronounced anxiety (2). Similarly, in a mixed sample of women with low-weight AN, partially recovered AN (90-120% expected body weight [EBW]), and healthy controls, we previously found an association between low fasting oxytocin levels and increased symptoms of alexithymia independent of BMI and estrogen status (11). Finally, in individuals with AN, oxytocin receptor polymorphisms were found to be associated with severity of ED pathology, and oxytocin and oxytocin receptor polymorphisms showed associations with disorder-specific decrements in emotion perception ability (12, 13), further pointing towards a potential involvement of the oxytocin system in AN psychopathology. While establishing a clinically relevant oxytocin-deficient state in AN, most investigations to date have examined oxytocin levels in AN without distinguishing between restricting and binge/purge subtypes, and the few studies that compared oxytocin levels across AN subtypes did not investigate their associations with type and severity of psychopathology within each AN presentation (2, 5, 7, 10, 11, 14, 15). One small study showed low levels of oxytocin in cerebrospinal fluid (CSF) in individuals with AN restricting type (n=5) but not those with AN binge/purge type (n=12) compared to healthy controls (n=11) (7). Other studies detected no differences in oxytocin levels between subtypes when assessed peripherally (14, 15). Of note, peripheral oxytocin levels have been shown to correlate with CSF levels, however current evidence indicates that this relationship may be context-dependent (16–19). To our knowledge, no studies have analyzed the correlation between oxytocin levels and psychopathology in different AN presentations, which could have clinical implications.

We aimed to extend our understanding of the role of oxytocin in the pathophysiology of AN and its different presentations by investigating the relationship between fasting peripheral oxytocin levels and severity of psychopathology, including eating disorder (ED) psychopathology, depressive and anxiety symptoms, and deficits in socioemotional functioning. Using a transdiagnostic approach, we recruited women with AN (BMI<18.5) and atypical AN (BMI≥18.5) who presented with active primarily restricting behaviors (AN/AtypAN-R) and those who were restricting combined with bingeing and/or purging behaviors (AN/AtypAN-BP). Based on the preliminary finding that, compared to healthy controls, CSF oxytocin levels were low in individuals with AN of the restricting type but not those with the binge/purge subtype (7), we hypothesized that lower fasting serum oxytocin levels would be associated with more pronounced ED psychopathology, depressive and anxiety symptoms, and deficits in socioemotional functioning in women with AN/AtypAN-R but not those with AN/AtypAN-BP.

## 2 Material and methods

### 2.1 Participants

Fifty-three females, 18-49 years, who met DSM-5 criteria for AN (n=31) or atypical AN (n=22) and participated in a randomized, placebo-controlled clinical trial of low-dose testosterone therapy for AN (clinicaltrials.gov identifier: NCT01121211) or an observational study of neurobiological underpinnings of illness trajectories in a sample of adolescent and young adult females with low-weight eating disorders (R01 MH103402), all of them with active AN or atypical AN at the time of data collection, were included in this study. Clinical characteristics, including endocrine parameters from partially overlapping datasets have been previously published (14, 20–23). However, the relationship between oxytocin levels and psychopathology, the focus of this paper, has not been reported. Binge/purge behaviors were defined by the occurrence of at least three behaviors over the past three months (frequency: ≥1/month). Of the 31 women with AN, 12 met criteria for AN/AtypAN-R, and 19 met criteria for AN/AtypAN-BP. Of the 22 participants with atypical AN, nine were categorized as AN/AtypAN-R and 13 as AN/AtypAN-BP. Participants were recruited from the community through advertisements and referrals from healthcare providers.

Exclusion criteria included a history of psychotic disorder, active suicidal ideation, diabetes mellitus, untreated hypothyroidism, unstable medical illness, pregnancy, breastfeeding, and low serum potassium levels. For participants of the clinical trial, further exclusion criteria (relevant to the trial) included free testosterone levels above the median for healthy women of reproductive age, use of androgens/androgen precursors over the past three months, not willing to use contraception, substance use disorder in the past six months, bipolar I disorder, severe current depressive symptoms (Hamilton Depression Rating Scale [HAM-D] (24) score >20, excluding two eating/weight loss items related to AN symptoms), investigational psychotropic drug within the past three months, dose or drug change in psychotropic treatment within the last six weeks, dose change in oral contraceptive pill or transdermal estrogen therapy within the last month, creatinine level >1.5x upper limit, or ALT >2x upper limit of normal. For participants of the observational study, the following additional exclusion criteria applied: other medical explanation for low weight, use of systemic hormones within eight weeks, use of Depo-Provera within three months, substance use disorder within the past month, hematocrit <30%, and gastrointestinal tract surgery.

### 2.2 Procedures

Study visits took place at the Massachusetts General Hospital Translational Clinical Research Center and the Athinoula A. Martinos Center for Biomedical Imaging. A screening visit to determine eligibility included the medical history, physical examination [with height, weight, frame size, calculation of BMI and %EBW using the Metropolitan Height and Weight Tables 1983 (25)], psychiatric interviews, questionnaires, and blood and urine collection. DSM-5 criteria for AN/Atypical AN were confirmed by the Structural Clinical Interview for DSM-IV (SCID-IV (26); clinical trial) or Eating Disorder Examination (EDE (27); observational study).

At the main study visit after an overnight fast, a urine pregnancy test and a morning blood draw for oxytocin and estradiol levels were obtained, and participants completed questionnaires to assess psychopathology. For participants of the observational study, the Toronto Alexithymia Scale (TAS-20) was completed within a week of that day. For participants enrolled in the clinical trial, all assessments were completed prior to randomization to the treatment arms and receipt of any study medication.

### 2.3 Self-report measures of psychopathology

#### 2.3.1 ED psychopathology

The Eating Disorder Examination – Questionnaire (EDE-Q) is a well-validated 28-item self-report measure that assesses attitudes and behaviors related to eating patterns and body image over the past 28 days and yields a global score and four subscale scores (Dietary Restraint, Eating Concern, Shape Concern, and Weight Concern). Scale scores range from 0 to 6 with higher scores representing more severe symptoms. Internal consistency for the Global Score is (α=0.90) (28). We considered an EDE-Q Global Score >2.5 (1 SD above the healthy population mean) to indicate active ED psychopathology (29–33).

#### 2.3.2 Depressive and anxiety symptoms

The 21-item Beck Depression Inventory-IA (BDI), a revised version of the original BDI (34), assesses severity of depressive symptoms over the previous two weeks with scores of 0-9 indicating minimal depressive symptoms, 10-16 mild depression, 17-29 moderate depression, and 30-63 severe depression (34). Internal consistency ranges from 0.73 to 0.92 (35).

The 20-item State-Trait Anxiety Inventory (STAI) Trait scale assesses trait anxiety with high internal consistency (α≥0.89) (36). In a female population (19-39 years), the mean STAI Trait Score was 36.2 with a standard deviation of 9.5 (36). STAI Trait Scores 1 SD above the mean are considered to be consistent with clinically significant anxiety symptoms (37).

#### 2.3.3 Socioemotional functioning

The 20-item TAS-20 is a well-validated measure of alexithymia with good internal consistency (α=0.81) (38, 39). Sum scores are determined for three subscales (Difficulty Identifying Feelings, Difficulty Describing Feelings, and Externally Oriented Thinking) together with a global score (≤51: nonalexithymia, 52–60: possible alexithymia, ≥61: alexithymia) (38). To capture the multifacetedness of socioemotional functioning, participants additionally completed the Liebowitz Social Anxiety Scale (LSAS-SR), the Dimensional Assessment of Personality Pathology – Basic Questionnaire (DAPP-BQ), and the Interpersonal Support Evaluation List (ISEL). The LSAS-SR assesses fear and avoidance of eleven social situations and 13 situations of public performance over the past week, which are summarized on four scales with higher scores indicating more severe psychopathology: Public Fear, Social Fear, Public Avoidance, and Social Avoidance (40). From the DAPP-BQ, participants rated 14 Suspiciousness and 16 Insecure Attachment items. Summated scores for Suspiciousness and Insecure Attachment scales were calculated with higher scores indicating more severe psychopathology (41). The 40-item ISEL assesses the perceived availability of potential social resources yielding a summary score lower scores indicating less perceived support (42).

### 2.4 Biochemical analysis

Serum samples were stored at -80°C and run in a single batch. Oxytocin concentration was measured in unextracted serum by ELISA in the Brigham Research Assay Core (BRAC) Laboratory using reagents purchased from Enzo Life Sciences, Farmingdale, NY, USA. We have previously demonstrated a robust correlation between extracted and unextracted serum oxytocin levels (43). The assay had a detection limit of 15 pg/mL. In-house quality-control samples had a mean of 81 and 120 pg/mL, and a low and high quality-control pools between-assay coefficient of variation (CV) of 18 and 20%, respectively. The cross-reactivity of Lys8-vasopressin, Arg8-vasopressin, met-enkephalin, VIP, somatostatin, Ser4, Ile8-oxytocin, and alpha-ANP in the oxytocin assay is <0.02%. Serum estradiol was measured by the BRAC using liquid chromatography-tandem mass spectrometry. The assay had a lower limit of detection of 1 pg/mL and intra-assay CV <5%.

### 2.5 Data analysis

STATA^®^ software (version 14.2; StataCorp LLC, College Station, TX, USA) was used for statistical analyses. Data were tested for normality using the Shapiro-Wilk test. Age, duration of illness, estradiol levels, and oxytocin levels were not normally distributed. Log-transformation prior to analysis resulted in a normal distribution for estradiol and oxytocin levels. For the other two measures, non-parametric tests were performed. Primary outcomes were EDE-Q Global, BDI Total, and STAI Trait scores for ED-specific, depressive, and anxiety symptoms, respectively. For socioemotional functioning, the TAS-20 served as the primary assessment tool. We have previously shown that among the TAS-20 scores, the Difficulty Identifying Feelings Score showed the strongest link with oxytocin levels (11). Accordingly, we chose the TAS-20 Difficulty Identifying Feelings Score as the primary outcome measure for socioemotional functioning in this study. Further TAS-20 scores and other measures of key subcomponents of socioemotional functioning (i.e., LSAS-SR, DAPP-BQ, and ISEL) were analyzed as additional exploratory outcomes.

We compared AN/AtypAN-R and AN/AtypAN-BP groups using t-tests for independent samples for continuous variables (except for age and duration of illness, for which Mann-Whitney U-tests were performed) and Fisher’s exact tests for nominal data. Pearson correlations investigated the relationship between (log-transformed) oxytocin levels and measures of psychopathology. In addition, we performed multivariate linear regression analyses to determine the relationship between baseline oxytocin levels and psychopathology controlling for time since diagnosis, which differed between study groups. Individuals with AN and atypical AN were combined for all analysis due to comparable characteristics (see **Table 1** for a comparison of hormone levels and key psychopathology endpoints). Statistical significance was defined as a two-tailed p-value <0.05. Data are reported as mean ± SD, median (IQR), or n (%).

**Table 1:** Participant characteristics of hormone levels and key psychopathology endpoints for women with anorexia nervosa (AN) versus atypical AN (AtypAN).

Characteristic	AN (n=31)	AtypAN (n=22)	p	Hedges’ g
Estradiol (pg/mL)^a^	55.9 ± 61.1	65.8 ± 48.8	n/a	n/a
Ln-estradiol^a^	3.3 ± 1.4	3.7 ± 1.2	0.364	-0.30
Fasting oxytocin (pg/mL)	1,018 ± 582	872 ± 308	n/a	n/a
Ln-fasting oxytocin	6.8 ± 0.5	6.7 ± 0.4	0.554	0.17
EDE-Q Global Score^b^	3.0 ± 1.6	3.5 ± 1.3	0.358	-0.33
BDI Total Score^c^	22.1 ± 10.8	23.4 ± 3.0	0.717	-0.15
STAI Trait Score	53.7 ± 10.4	54.1 ± 13.1	0.906	-0.03
TAS-20 Difficulty Identifying Feelings Score^d^	20.0 ± 6.5	20.8 ± 6.6	0.699	-0.12

Mean ± SD. ^a^Data available for 36 participants (21 with AN and 15 with AtypAN), all of whom were off oral contraceptive pill medication. ^b^Data available for 35 participants (19 with AN and 16 with AtypAN). ^c^Data available for 36 participants (20 with AN and 16 with AtypAN). ^d^Data available for 50 participants (29 with AN and 21 with AtypAN). BDI, Beck Depression Inventory-IA; EDE-Q, Eating Disorder Examination Questionnaire; STAI, State-Trait Anxiety Inventory; TAS-20, Toronto Alexithymia Scale.

## 3 Results

### 3.1 Participant characteristics

Participant characteristics are presented in **Table 2**. Time since diagnosis was shorter in the AN/AtypAN-R group compared to the AN/AtypAN-BP group, while age, BMI, and estrogen status did not differ between groups. Furthermore, AN/AtypAN-R and AN/AtypAN-BP groups showed no difference in frequency of key comorbidities and medication intake.

**Table 2:** Participant characteristics for women with anorexia nervosa (AN)/Atypical AN who are solely restricting (AN/AtypAN-R) versus those who restrict in combination with binge/purge behaviors (AN/AtypAN-BP).

Characteristic	AN/AtypAN-R (n=21)	AN/AtypAN-BP (n=32)	p	Effect size^a^
Age (years)	25.0 (21.0-28.0)	21.5 (19.5-33.0)	0.636	r=0.06
Duration of illness (months)^b^	8.0 (5.0-13.0)	16.5 (11.0-27.5)	**0.004**	r=-0.41
Lowest adult weight (kg)	43.8 ± 5.8	42.8 ± 5.8	0.533	g=0.17
Weight (kg)	49.9 ± 4.9	48.7 ± 5.4	0.417	g=0.23
BMI (kg/m^2^)	18.5 ± 1.9	18.2 ± 1.4	0.548	g=0.18
%EBW	83.9 ± 6.9	84.7 ± 7.5	0.730	g=-0.11
Amenorrhea^c^	6 (30.0)	9 (28.1)	1.000	OR=1.10
Current MDD	10 (47.6)	17 (53.1)	0.782	OR=0.80
Current GAD	15 (71.4)	19 (59.4)	0.400	OR=1.71
Current OCD	1 (4.8)	1 (3.1)	1.000	OR=1.55
Current PTSD	6 (28.6)	7 (21.9)	0.746	OR=1.43
Antidepressant medication	14 (66.7)	20 (62.5)	1.000	OR=1.20
Anxiolytic medication	10 (47.6)	11 (34.4)	0.397	OR=1.74
Mood stabilizers	1 (4.8)	3 (9.4)	1.000	OR=0.48
Antipsychotic medication	4 (19.1)	3 (9.4)	0.415	OR=2.27
Hypnotic medication	1 (4.8)	3 (9.4)	1.000	OR=0.48
Melatonin	2 (9.5)	0 (0.0)	0.152	N/A
OCPs^c^	8 (40.0)	7 (21.9)	0.213	OR=2.38
Estradiol (pg/mL)^d^	76.6 ± 58.1	51.7 ± 53.8	n/a	n/a
Ln-estradiol^d^	3.9 ± 1.3	3.3 ± 1.3	0.229	g=0.42
Fasting oxytocin (pg/mL)	873 ± 534	1,013 ± 456	n/a	n/a
Ln-oxytocin	6.6 ± 0.5	6.8 ± 0.4	0.158	g=-0.41
EDE-Q Global Score^e^	2.8 ± 1.6	3.9 ± 1.1	**0.025**	g=-0.75
BDI Total Score^f^	19.3 ± 11.3	27.3 ± 9.5	**0.032**	g=-0.74
STAI Trait Score	51.5 ± 11.7	55.4 ± 11.2	0.231	g=-0.34
TAS-20 Difficulty Identifying Feelings Score^g^	19.0 ± 6.9	21.3 ± 6.1	0.216	g=-0.35
TAS-20 Difficulty Describing Feelings Score^g^	15.1 ± 4.9	16.9 ± 4.5	0.185	g=-0.38
TAS-20 Externally Oriented Thinking Score^g^	17.9 ± 4.9	18.6 ± 4.7	0.606	g=-0.14
TAS-20 Total Score^g^	52.0 ± 13.1	56.9 ± 11.2	0.168	g=-0.40
LSAS-SR Social Fear Score^f^	14.7 ± 6.3	16.9 ± 5.2	0.261	g=-0.37
LSAS-SR Public Fear Score^f^	15.6 ± 6.4	19.4 ± 4.9	0.064	g=-0.64
LSAS-SR Social Avoidance Score^f^	13.4 ± 7.2	16.3 ± 6.4	0.218	g=-0.41
LSAS-SR Public Avoidance Score^f^	13.0 ± 7.1	16.7 ± 6.6	0.132	g=-0.52
DAPP-BQ Suspiciousness Score^f^	27.8 ± 8.9	34.4 ± 11.7	0.061	g=-0.64
DAPP-BQ Insecure Attachment Score^f^	36.3 ± 14.1	42.9 ± 16.2	0.207	g=-0.43
ISEL Total Score^f^	82.8 ± 17.8	68.9 ± 22.1	**0.045**	g=0.69

Mean ± SD, median (IQR), or n (%). Significant values (p<0.05) are highlighted in bold. ^a^Effect sizes are reported as Hedges’ g for group comparisons of normally distributed continuous variables analyzed with independent-sample t-tests, r for non-normally distributed variables analyzed with Mann-Whitney U-test, and OR (exact) for nominal variables analyzed with Fisher’s exact test. ^b^Four participants with AN/AtypAN-BP did not provide information for duration of illness. ^c^Data available for 52 participants (20 participants with AN/AtypAN-R and 32 with AN/AtypAN-BP). ^d^Data available for 36 participants, all of whom were off OCP medication (12 participants with AN/AtypAN-R and 24 with AN/AtypAN-BP). ^e^Data available for 35 participants (21 participants with AN/AtypAN-R and 14 with AN/AtypAN-BP). ^f^Data available for 36 participants (21 participants with AN/AtypAN-R and 15 with AN/AtypAN-BP). ^g^Data available for 50 participants (21 participants with AN/AtypAN-R and 29 with AN/AtypAN-BP). BDI, Beck Depression Inventory-IA; BMI, body mass index; DAPP-BQ, Dimensional Assessment of Personality Pathology – Basic Questionnaire; %EBW, percent expected body weight; EDE-Q, Eating Disorder Examination Questionnaire; GAD, generalized anxiety disorder; ISEL, Interpersonal Support Evaluation List; LSAS-SR, Liebowitz Social Anxiety Scale; MDD, major depressive disorder; OCD, obsessive-compulsive disorder; OCPs, oral contraceptive pills; PTSD, posttraumatic stress disorder; STAI, State-Trait Anxiety Inventory; TAS-20, Toronto Alexithymia Scale.

### 3.2 Self-report measures of psychopathology

Group means and between-group comparisons of psychopathology are summarized in **Table 2**. Twelve participants with AN/AtypAN-R (57.1%) and 11 participants with AN/AtypAN-BP (73.3%) had an EDE-Q Global Score in the clinical range. Eleven participants with AN/AtypAN-R (52.4%) and 13 individuals with AN/AtypAN-BP (86.7%) had a BDI Total Score consistent with moderate or severe depressive symptoms. Fifteen participants with AN/AtypAN-R (71.4%) and 26 individuals with AN/AtypAN-BP (81.3%) had a STAI Trait Score consistent with clinically significant anxiety. Nine individuals with AN/AtypAN-R (42.9%) and 19 participants with AN/AtypAN-BP (65.5%) had a TAS-20 Total Score in the range of possible or definite symptoms of alexithymia. ED psychopathology and depressive symptoms were more pronounced in individuals with AN/AtypAN-BP than in those with AN/AtypAN-R, as indicated by higher EDE-Q Global and BDI Total scores, respectively. In addition, the AN/AtypAN-BP group had a lower perception of social support than the AN/AtypAN-R group, as indicated by a lower ISEL Total Score. When controlling for illness duration, no significant group differences remained (ps≥0.094).

### 3.3 Oxytocin levels and relationship with psychopathology

**Figure 1:**
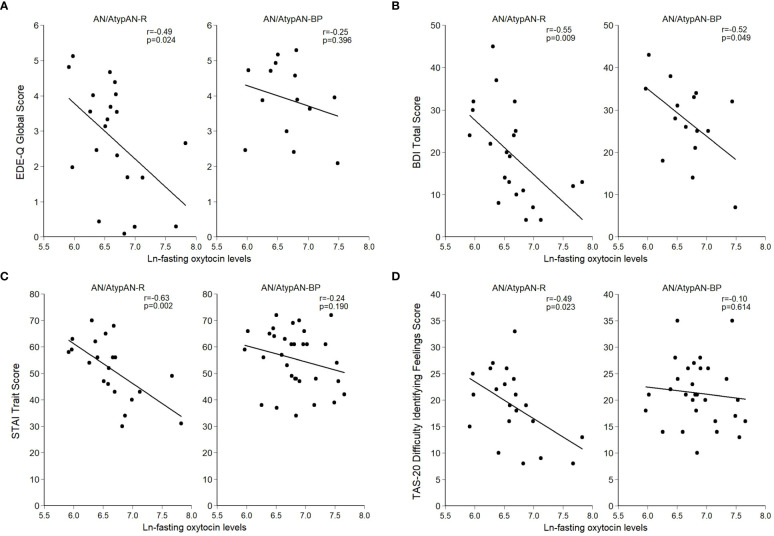
Relationship between (log-transformed) fasting oxytocin levels and psychopathology in women with anorexia nervosa (AN)/Atypical AN who are solely restricting (AN/AtypAN-R) and those who restrict in combination with binge/purge behaviors (AN/AtypAN-BP). **(A)** Eating Disorder Examination – Questionnaire (EDE-Q) Global Score (eating disorder psychopathology); **(B)** Beck Depression Inventory-IA (BDI) Total Score (depressive symptoms); **(C)** State-Trait Anxiety Inventory (STAI) Trait Score (anxiety symptoms); and **(D)** Toronto Alexithymia Scale (TAS-20) Difficulty Identifying Feelings Score (socioemotional functioning).

Fasting oxytocin levels did not differ between groups (**Table 2**). In individuals with AN/AtypAN-R, oxytocin levels were broadly associated with symptom severity, namely, lower oxytocin levels were associated with higher EDE-Q Global, BDI Total, and STAI Trait scores, reflecting more pronounced ED, depressive, and anxiety symptoms, respectively (**Table 3**; **Figure 1**). Furthermore, in individuals with AN/AtypAN-R, oxytocin levels were related to socioemotional functioning with lower oxytocin levels being associated with higher TAS-20 Difficulty Identifying Feelings, LSAS-SR Social Fear, LSAS-SR Public Fear, LSAS-SR Social Avoidance, and DAPP-BQ Suspiciousness scores (indicating more pronounced deficits in socioemotional functioning) and decreased ISEL Total Scores (indicating a reduced perception of social support). Conversely, in individuals with AN/AtypAN-BP, the only observed association was between low oxytocin levels and higher BDI Total Scores; no other relationships reached significance in the AN/AtypAN-BP group (**Table 3**; **Figure 1**).

**Table 3:** Associations between (log-transformed) fasting oxytocin levels and psychopathology in women with anorexia nervosa (AN)/Atypical AN who are solely restricting (AN/AtypAN-R) versus those who restrict in combination with binge/purge behaviors (AN/AtypAN-BP).

Self-report measures of psychopathology	AN/AtypAN-R (n=21)		AN/AtypAN-BP (n=32)	
	r	p	r	p
Primary endpoints				
EDE-Q Global Score (eating disorder psychopathology)	-0.49	**0.024**	-0.25^a^	0.396
BDI Total Score (depressive symptoms)	-0.55	**0.009**	-0.52^b^	**0.049**
STAI Trait Score (anxiety symptoms)	-0.63	**0.002**	-0.24	0.190
TAS-20 Difficulty Identifying Feelings Score (socioemotional functioning)	-0.49	**0.023**	-0.10^c^	0.614
Exploratory endpoints (socioemotional functioning)				
TAS-20 Difficulty Describing Feelings Score	-0.29	0.205	0.04^c^	0.834
TAS-20 Externally Oriented Thinking Score	-0.09	0.684	0.34^c^	0.075
TAS-20 Total Score	-0.40	0.071	0.11^c^	0.586
LSAS-SR Social Fear Score	-0.56	**0.008**	-0.15^b^	0.582
LSAS-SR Public Fear Score	-0.44	**0.046**	-0.21^b^	0.457
LSAS-SR Social Avoidance Score	-0.57	**0.006**	-0.07^b^	0.814
LSAS-SR Public Avoidance Score	-0.41	0.063	-0.02^b^	0.932
DAPP-BQ Suspiciousness Score	-0.49	**0.024**	-0.16^b^	0.562
DAPP-BQ Insecure Attachment Score	-0.25	0.285	0.14^b^	0.618
ISEL Total Score	0.47	**0.031**	0.51^b^	0.051

Significant values (p<0.05) are highlighted in bold. ^a^Based on 14 participants. ^b^Based on 15 participants. ^c^Based on 29 participants. BDI, Beck Depression Inventory-IA; DAPP-BQ, Dimensional Assessment of Personality Pathology – Basic Questionnaire; EDE-Q, Eating Disorder Examination – Questionnaire; ISEL, Interpersonal Support Evaluation List; LSAS-SR, Liebowitz Social Anxiety Scale; STAI, State-Trait Anxiety Inventory; TAS-20, Toronto Alexithymia Scale.

## 4 Discussion

To the best of our knowledge, this is the first study to examine relationships between fasting serum oxytocin levels and psychopathology in a broad sample of individuals with active AN and atypical AN who engage in primary restriction (AN/AtypAN-R) and, separately, in those who restrict combined with binge and/or purge behaviors (AN/AtypAN-BP). In females with AN/AtypAN-R, we observed robust negative correlations between basal oxytocin levels and severity of ED psychopathology, depressive and anxiety symptoms, and impairment of socioemotional functioning. In contrast, in women with AN/AtypAN-BP, there was only an association between lower oxytocin levels and more pronounced depressive symptoms, while no other significant relationships between oxytocin levels and psychopathology were identified in this study. These data indicate possible differences in underlying pathophysiology across AN presentations.

Our findings suggest a role of oxytocin pathways in the ED pathology of AN/AtypAN-R. The result pattern observed in the AN/AtypAN-R group resembles findings we previously reported in individuals with AN in partial recovery, where fasting oxytocin levels were associated with greater ED pathology, and individuals with clinically significant ED pathology displayed lower oxytocin levels than those without clinically significant symptoms (2). While the previous study did not distinguish between individuals with primarily restricting and those with additional binge/purge presentation, in the present investigation in women with AN and atypical AN, despite similar levels of serum oxytocin in females with AN/AtypAN-R and AN/AtypAN-BP, we found a robust relationship between oxytocin and ED psychopathology in individuals with AN/AtypAN-R but not AN/AtypAN-BP. The lack of an observed relationship between oxytocin and psychopathology in women with AN/AtypAN-BP could be the result of binge/purge behaviors altering peripheral oxytocin, and it is still possible that central oxytocin and psychopathology are related in this AN presentation. Alternatively, it is conceivable that oxytocin plays a more substantial role in the modulation of psychopathology in AN/AtypAN-R than AN/AtypAN-BP. While future research studies are needed to understand the cause of the different observation of oxytocin levels in CSF, serum oxytocin appears to be a biomarker for severity of psychopathology specifically in AN/AtypAN-R.

The potential relevance of oxytocin in the psychopathology of AN/AtypAN-R seems to go beyond ED pathology and also spans depressive and anxiety symptoms and socioemotional functioning. Comorbid depression and anxiety are common in AN (44, 45). On average, our sample showed moderate levels of depressive symptoms and clinically significant anxiety. Preclinical and clinical studies have demonstrated that oxytocin has antidepressant and anxiolytic properties, including improving psychopathology and pathophysiology in clinical populations (46–49). For example, single-dose intranasal administration of oxytocin reduced amygdala reactivity and functional connectivity to fear-inducing stimuli in individuals with generalized social anxiety disorders (50, 51), and repeated doses of intranasal oxytocin over four weeks added to pharmacological treatment with escitalopram improved depressive symptoms in individuals with treatment-resistant major depressive disorders (52). In women with partially recovered AN, we previously reported a correlation between lower fasting serum oxytocin levels and more pronounced anxiety symptoms (2). For the first time, the present study reports a relationship between low levels of oxytocin and more pronounced depressive and anxiety symptoms in individuals with active AN/AtypAN-R, suggesting that low oxytocin may mediate mood and anxiety symptoms in this subgroup. In individuals with active AN/AtypAN-BP, we also found a negative association between oxytocin levels and depressive symptoms, mirroring the pattern in AN/AtypAN-R, whereas there was no evidence for a linear relationship between oxytocin levels and anxiety in AN/AtypAN-BP.

In addition to a negative relationship between oxytocin levels and difficulty identifying feelings in females with AN/AtypAN-R but not AN/AtypAN-BP, our study provides a multifaceted exploratory assessment of socioemotional functioning that shows a consistent pattern of lower oxytocin levels associated with worse socioemotional functioning in women with AN/AtypAN-R but not AN/AtypAN-BP. Individuals with AN often show impaired socioemotional functioning by means of increased social anxiety (53), suspiciousness and insecure attachment (54), difficulty recognizing others’ emotions (55), and alexithymia (56), which often does not resolve with weight gain (54, 57, 58). Animal research has demonstrated a prosocial role of oxytocin, including the promotion of maternal and pair bonding (59, 60), approach behavior under stress (61), and increased duration of eye contact and higher number of prosocial choices in rhesus macaques (62). In humans, oxytocin administration has been shown to improve socioemotional functioning in healthy individuals (63) and across a variety of psychiatric conditions associated with socioemotional challenges, including autism spectrum disorder, schizophrenia, and social anxiety (50, 63, 64). In the context of AN, a previous study from our group of women with low-weight AN (without distinction between presentations), partially recovered AN (90-120% EBW), and healthy controls found an association between low fasting oxytocin levels and increased symptoms of alexithymia independent of BMI and estrogen status, raising the question of whether low oxytocin levels could contribute to social emotional functioning difficulties in AN (11). However, relationships between oxytocin levels and other measures of socioemotional functioning were not identified, groups were analyzed conjointly rather than separately, and the role of AN presentations was not addressed. Some studies have suggested closer resemblance of the binge/purge presentation of AN to bulimia nervosa than primarily restricting AN (7, 15). Our study extends our prior findings by showing broad and consistent relationships between oxytocin levels and socioemotional functioning with lower oxytocin levels being associated with more pronounced socioemotional dysfunction in women with AN/AtypAN-R but not AN/AtypAN-BP. The broader sample comprising individuals with AN and atypical AN increases generalizability of the observed findings.

Limitations of this study include the relatively small sample size, which could have introduced bias. Furthermore, as a cross-sectional investigation we report associations and cannot determine causality. Longitudinal studies in larger samples of women with AN/AtypAN-R and AN/AtypAN-BP that build on the presented findings and further explore the role of additional key characteristics will be essential to further investigate the role of oxytocin in mediating psychopathology. For example, a longer duration of illness in individuals with AN/Atypical AN-BP compared to AN/Atypical AN-R represents a commonly observed difference. This difference is rooted in the fact that diagnostic crossover during prolonged illness from AN/Atypical presentations with primarily dietary restriction to eating disorders featuring binge-eating and/or purging is common (~50%), while the reverse crossover rarely occurs (65). A lack of an observed relationship between oxytocin and psychopathology in our sample of individuals with AN/AtypAN-BP could be related to a longer duration of illness, and/or it could highlight a neurobiological shift that occurs simultaneously with and/or is driving the observed behavioral changes taking place with diagnostic crossover. Future studies should examine groups of individuals with AN/Atypical AN-R and AN/Atypical AN-BP who are comparable in duration of illness to shed light on the separate and joint impact of symptom presentation and illness duration on the studied endocrine-psychopathological link. Furthermore, follow-up prospective studies in individuals undergoing diagnostic crossover from AN/AtypAN-R to AN/AtypAN-BP are needed to build on the reported findings and examine the relationship between oxytocin levels and psychopathology longitudinally to better understand its potential role in diagnostic crossover. Lastly, future studies investigating the relationship between oxytocin levels and psychopathology across AN subtypes should consider including CSF oxytocin levels to better understand the relationship between central and peripheral oxytocin levels in the context of these research questions.

In summary, the present study is the first to show consistent relationships between fasting serum oxytocin levels and psychopathology (spanning ED psychopathology, depressive and anxiety symptoms, and impairments in socioemotional functioning) in women with active AN/AtypAN-R. Our sample of individuals with active AN/AtypAN-BP only showed an association between low oxytocin levels and depressive symptoms, while no other relationships between oxytocin and psychopathology were observed. These findings are crucial to better elucidate oxytocin physiology and its role in psychopathology in AN presentations, highlighting a potentially different underlying psychopathology in AN-R and AN-BP. Additional studies are needed to further investigate the role of oxytocin in the psychopathology of AN and explore the potential of oxytocin pathways as neurohormonal treatment targets for selected AN presentations, and future randomized controlled trials could consider using the outcomes that we found to be associated with peripheral oxytocin levels as primary endpoints.

## Data availability statement

Data from the randomized, placebo-controlled clinical trial of low-dose testosterone therapy for AN (clinicaltrials.gov identifier: NCT01121211) are available upon reasonable request to KM (kkmiller@mgh.harvard.edu). Data from the observational study of neurobiology of low-weight eating disorders (R01 MH103402) will be available upon request to the corresponding author and through National Institute of Mental Health.

## Ethics statement

This research was approved by the Institutional Review Board of Mass General Brigham and carried out in accordance with the Declaration of Helsinki. Informed written consent was obtained from all participants. 

## Author contributions

FP, FG, AA, and EL conceived the study idea and designed this investigation. FP and AA performed the analysis. FP, FG, and EL wrote the manuscript. KE, MM, KM, AK, and AA provided feedback on the manuscript. All authors contributed to the article and approved the submitted version.
